# Gene–Lifestyle Interactions in Obesity

**DOI:** 10.1007/s13668-012-0022-2

**Published:** 2012-06-26

**Authors:** Jana V. van Vliet-Ostaptchouk, Harold Snieder, Vasiliki Lagou

**Affiliations:** 1 Department of Endocrinology, University Medical Center Groningen, University of Groningen, Groningen, The Netherlands; 2 Unit of Genetic Epidemiology and Bioinformatics, Department of Epidemiology, University Medical Center Groningen, University of Groningen, Groningen, The Netherlands; 3 Oxford Centre for Diabetes, Endocrinology, and Metabolism and Wellcome Trust Centre for Human Genetics, University of Oxford, Roosevelt Drive, Oxford, OX3 7BN UK

**Keywords:** Gene–environment interaction, Lifestyle, Genetics, Environment, Epigenetics, Obesity, Epigenome

## Abstract

Obesity is a complex multifaceted disease resulting from interactions between genetics and lifestyle. The proportion of phenotypic variance ascribed to genetic variance is 0.4 to 0.7 for obesity and recent years have seen considerable success in identifying disease-susceptibility variants. Although with the advent of genome-wide association studies the list of genetic variants predisposing to obesity has significantly increased the identified variants only explain a fraction of disease heritability. Studies of gene–environment interactions can provide more insight into the biological mechanisms involved in obesity despite the challenges associated with such designs. Epigenetic changes that affect gene function without DNA sequence modifications may be a key factor explaining interindividual differences in obesity, with both genetic and environmental factors influencing the epigenome. Disentangling the relative contributions of genetic, environmental and epigenetic marks to the establishment of obesity is a major challenge given the complex interplay between these determinants.

## Introduction

Obesity was once considered a problem of economically developed countries, but the number of overweight and obese people is now dramatically increasing in low- and middle-income countries at a rate never seen before [1]. If recent trends continue unabated, by 2030, the absolute numbers could rise to a total of 2.16 billion overweight and 1.12 billion obese individuals, or 38 % and 20 % of the world’s adult population, respectively [2].

As the fundamental cause of obesity and overweight (defined by anthropometric measures: body mass index [BMI], waist circumference [WC] and/or waist-to-hip ratio [WHR]) is an energy imbalance between calories consumed on the one hand and calories expended on the other hand, increases in rates of obesity must reflect a state of positive energy balance, which is very likely a result of the profound changes in society and in behavioral patterns of populations over recent decades. Indeed, it is widely accepted that multiple factors contribute to this epidemic, including economic growth, modernization, urbanization and, most importantly, changes in our lifestyle, as eating habits have shifted to greater consumption of energy-dense foods that are high in fats and sugars, while at the same time, physical activity has decreased [1, 3]. Although a healthy lifestyle could be the apparent remedy for obesity, its implementation in the general population has proven difficult so far. Given the fact that people respond differently to the “obesogenic” environment owing to genetic predisposition, understanding the causes and pathophysiology of obesity is very important for prevention and therapy. Even in the presence of a strong “obesogenic” environment, hereditary factors remain key contributors to the disease etiology. Ethnic/racial differences in obesity even in comparable environments [4, 5] indicate that obesity is most likely the result of a complex interplay between multiple genetic, behavioral, social and environmental factors that affect energy balance and, thus, body weight regulation [6–9].

Over the past two decades, numerous strategies have been employed for the identification of genetic determinants of obesity, including studies of severe forms of obesity, genome-wide linkage studies, candidate gene analyses and genome-wide association studies (GWAS). Since 2005, the novel GWAS approach has led to breakthrough progress in our understanding of the genetic determinants of common obesity. Almost 50 loci have been identified and are collectively reported in the National Human Genome Research Institute GWAS catalogue (http://www.genome.gov/gwastudies/) [10]. Among those GWAS findings, the first obesity susceptibility locus identified was the *FTO* gene, which has the largest effect on obesity risk to date; each additional risk allele in *FTO* was shown to be associated with a 1- to 1.5-kg increase in body weight and a 20 % to 30 % increase in obesity risk [11, 12]. Since it is widely assumed that gene–environment interaction (GEI) must have an effect on adiposity, several epidemiologic studies have explored the relationship between lifestyle and obesity susceptibility genes, reporting significant interactive effects. Despite discrepancies in the reported results, some new insights into the role of gene–lifestyle interaction in obesity have been obtained. In the current review, we evaluate the recent successes in the examination of GEI in obesity and describe the main findings. We then examine the machinery that underlies GEI in obesity, focusing on epigenetics and particularly DNA methylation as a mechanism for these interactions. Finally, we discuss the challenges of the existing and emerging approaches in studying GEI.

## Genetic Determinants of Obesity

Until recently, progress in finding obesity-susceptibility genes was rather slow. Numerous groups have been involved in research related to the genetics of common obesity, with a major focus on candidate gene studies. Those genes were selected based on their known functional role in physiologic pathways (e.g. regulating body weight or energy metabolism) [13]. Between 1996 and 2005, the Obesity Gene Map (http://obesitygene.pbrc.edu/) extensively evaluated all published results, including monogenic forms of obesity, transgenic and knockout animal models, quantitative trait loci from animal cross-breeding experiments, linkages from genome scans and candidate gene association studies [14]. In the last update of the Obesity Gene Map published in 2006, 127 candidate genes were reported, of which less than 20 % were replicated by 5 or more studies. Such a high level of non-replication was the result of many limitations of the candidate gene study approach, such as small sample size and, thus, insufficient statistical power to detect small effects, as well as lack of type 1 error control, among others [15•].

In the past few years, a novel approach (GWAS) that involves scanning of many thousands of samples using the latest advances in genotyping technology (i.e. high-density, genome-wide arrays to assay hundreds of thousands of single nucleotide polymorphisms [SNPs] that capture the majority of common variation in the human genome) have led to breakthrough progress in the identification of obesity-susceptibility genes. To date, large-scale meta-analyses of GWAS for overall and abdominal obesity along with a recent GWAS meta-analysis for percent body fat (%BF) have reported 32 genetic loci associated with BMI, 14 loci related to WHR and 2 loci for %BF (see Day and Loos [15•] for a recent overview of GWAS findings of obesity-related traits). The effect sizes of the 32 established BMI-associated loci ranged from 0.06 to 0.39 kg/m^2^ (or ~0.17 to ~1.13 kg for an adult of ~170 cm in height) per risk allele, with the *FTO* gene having the largest effect size [16]. For the 14 novel WHR loci, the effect sizes varied from 0.019 to 0.042 units per risk allele [17], while the risk alleles for the new %BF loci were associated with an increase ranging from 0.14 to 0.33 % in body fat [18]. Remarkably, the combined effect of all obesity-associated variants is very modest and explains less then 2 % of the BMI heritability [16]. Since the heritability of BMI is estimated to be relatively high (between 40 % and 70 % [15•]), the major question is: what accounts for the missing heritability? Among the suggested explanations is that the modifying effects of environmental factors on genetic predisposition to obesity might partially account for the unexplained interindividual variation in BMI [19••].

## Lifestyle Risk Factors for Obesity

Many specialists and scientists in the obesity research field agree that the dramatic increase in the prevalence of overweight and obesity over the past few decades is mainly attributable to the modern (Western) lifestyle, which is characterized by an excessive caloric intake and a sedentary lifestyle [1]. On the basis of many observational and epidemiologic studies, we currently know that the major environmental risk factors for obesity are unhealthy dietary habits (e.g. low in vegetables and fruits high in fat), decreased physical activity and alcohol consumption [20–25]. Overall and abdominal obesity show a negative association with such modifiable lifestyle habits as a Mediterranean-type diet, moderate alcohol consumption and daily physical activity [26]. However, all these well-established risk factors for obesity cannot explain a large proportion of the obesity cases, as there is a high interindividual susceptibility to weight gain in a common “obesogenic” environment. Thus, the most accepted point of view is that the modern obesity epidemic occurs due to a complex interplay between multiple genetic, behavioral and environmental factors. Recently, Speakman and colleagues [27•] suggested a new interesting model for body weight regulation to explain the mechanism underlying the current obesity epidemic. Briefly, this model suggests the presence of upper and lower boundaries defining the set points at which physiologic regulation of body weight and/or fatness becomes activated. While the distance between these intervention points is genetically determined, the changes in body weight depend on the prevailing direction of the environmental pressure (e.g. in the presence of an “obesogenic” environment with increased food supply driving up food intake, only some people become obese). The hypothesis provides a compelling explanation for the observed complexity of the obesity problem and integrates data and research from both the behavioral–nutritional and the molecular genetic–physiologic fields [27•].

## Studies on GEI in Obesity

It is well recognized that the investigation of GEI in obesity etiology has not been given sufficient attention in genetic studies [19••]. The majority of GWAS, in particular, have not examined GEI, mainly due to lack of data on environmental measurements [28]. GEI refers to the situation in which genotypes only have their effect in the context of an environment and environments have modifying effects that are dependent on genotypes. In other words, in the presence of the “obesogenic” environment, some individuals with a genetic predisposition to develop obesity will be more prone to gain weight compared with individuals with genetic “resistance” to obesity [29]. A growing body of recent evidence supports a significant role of GEI in obesity and related metabolic diseases [30–32, 33••]. To provide an overview on the most current publications in relationship to GEI in obesity, we searched PubMed (February 15, 2012) using a combination of keywords for genetic studies (i.e. gene, genetic variant, polymorphism, SNP), different obesity-related phenotypes (i.e. overweight, obesity, BMI, waist, hip, WHR, fat, adiposity) and environmental factors (i.e. feeding, diet, physical activity, alcohol, smoking, stress). This retrieved 522 papers published since January 1, 2011, of which 29 were selected as the most relevant to the present review.

The selected papers examined 1) candidate genes for obesity known to play a role in the functional pathways related to metabolic regulation and 2) novel obesity-susceptibility loci identified in recent GWAS. Two approaches were used to investigate the relationship between those genes and different lifestyle factors, such as dietary components, eating habits, physical activity, sedentary behavior and psychological stress: observational and intervention studies. The observational studies are relatively easy to perform. As soon as environmental exposures and genotyping information are collected, the GEIs are examined using cross-sectional or case–control designs. However, the major limitation of these designs is their inability to identify the individual and combined effects of the genetic and lifestyle risk factors or, in other words, to answer the question of *how* genetic predisposition and behavior combine to determine the risk of obesity [34]. Moreover, the observational studies are susceptible to multiple sources of bias (e.g. selection or recall bias) because environmental exposure and the outcome of interest are assessed simultaneously. In contrast, intervention study designs allow minimization of bias and provide direct control of the environmental factors by defining the experimental conditions a priori (e.g. a specific diet or level of physical activity). However, because these studies are usually small and short term, they have low statistical power and are not appropriate for investigating long-term effects [32].

### GEI Studies for Candidate Genes

Overall, the investigation of GEI for biological candidate genes has not been very successful and only a few findings for GEI in obesity were replicated in independent studies. This is the result of small effect sizes and very modest levels of significance for the majority of candidate genes proven to be associated with obesity [34]. Furthermore, as interaction effect sizes are likely to be of even smaller magnitude, many published small-scale reports of GEI for obesity were underpowered and, thus, are probably false positive [33••].

Since January 1, 2011, a few studies have reported GEI consistent with those from previous studies (Table 1 provides a summary of the most relevant GEI studies in obesity published during the past year) [30–32]. For example, variants in the β2-adrenergic receptor (*ADRB2*)- rs1042714 (Gln27Glu) and rs1042713 (Arg16Gly)- were associated with higher risk of obesity among the individuals with unhealthy lifestyles (i.e. smoking and reduced physically activity) [35, 36] and were shown to have a moderating effect on diet-induced changes on body weight and body composition [37]. Significant genotype–dietary fat interactions for obesity traits have also been reported for the apolipoprotein genes that regulate lipid metabolism (*APOA1, APOA2, APOA5*, *APOB)* [38–40], confirming previously observed GEI: the *APOA2*–saturated fat interaction on body weight and the protective effect of the *APO5*-1131 C minor allele on obesity in individuals on high-fat diets [39–41]. In addition, an association between *APOE* genotypes and increased BMI and WC dependent on psychological stress was reported in Danish men [42]. Also, the peroxisome proliferator-activated receptor-γ (*PPARγ*) gene, which has been extensively studied for GEI related to obesity and type 2 diabetes [32, 43], was reported to have a diet-related effect on risk to obesity with the Pro12 allele being associated with increased adiposity in a high-fat diet group [44]. In addition, the lactase (*LCT*) gene was shown to be associated with risk to obesity only in individuals who had high milk consumption [45]. Three additional observational studies investigated multiple candidate genes from metabolically relevant pathways: a few positive associations between genes, dietary components and obesity were observed (Table 1) [46–48].

**Table 1:** Selected gene–environment interaction studies on obesity for candidate genes

Gene (SNP)	Obesity phenotype	Lifestyle factor	Type of study	Population	Sample size	Major findings	Reference
*ADRB2* (Gln27Glu Arg16Gly)	BMI, body fat mass and lean mass	Energy-restricted diet	Intervention study	Spanish obese women	78	In response to a 12-wk energy-restricted diet, women carrying the Glu allele had a greater reduction in body weight and lost more lean mass than the non-Glu allele carriers	Ruiz et al. [37]
*ADRB2* (R16G)	BMI	Overeating, smoking, parent’s obesity	Observational, family-based study	Korean population	163 adolescents with parents	Smoking parents who overate and carried the Arg allele had an increased risk of obesity compared with nonsmoking parents who had none of these factors	Lee et al. [35]
*ADRB2, APOB, NOS3*	BMI, WC, BF%, VAT, SAT	PA, diet	Observational study	EA and AA adolescents (13–19 years)	621	Significant interactions were revealed between the *ADRB2* Arg16Gly SNP and vigorous PA on VAT, SAT and WC, suggesting that Gly16 homozygotes may benefit less from increased PA to reduce their weight	Lagou et al. [36]
*APOA1/C3/A4/A5* (12 SNPs)	BMI, WC	Dietary fat intake	Observational study	The Boston Puerto Rican Health Study	821	Significant interactions were observed between dietary fat intake and *APOA1-75* in association with WC. Homozygotes for the common allele of *APOA1-75, APOA4 N147S and APOA5* S19W had lower WC when consuming <31 % of total fat from energy than participants with the minor allele	Mattei et al. [39]
*APOA1* (rs670), *APOB* (rs512535)	BMI, WC	Dietary fat intake	Observational, case–control study	The LIPGENE-SU.VI.MAX study	1,754	Risk of metabolic syndrome was modified by dietary fat intake, whereby the deleterious effects conferred by GG homozygosity for *APOB* and *APOA1* were exacerbated among individuals consuming a high-fat diet, particularly high in MUFA. The GG homozygotes had greater BMI compared with A allele carriers and with the GG homozygotes with the lowest fat intake	Phillips [38]
*APOA2* (−265 T > C)	BMI	Saturated fat intake	Observational study	Mediterranean and multiethnic Asian populations	4,602	In Mediterranean individuals, the CC genotype was associated with a 6.8 % greater BMI in those consuming a high saturated fat diet; also, the CC genotype was significantly associated with higher obesity prevalence in Chinese and Asian Indians only with a high saturated fat intake	Corella et al. [41]
*APOA5* (−1131 T > C)	BMI	Dietary fat intake	Observational study	Spanish overweight and obese adults	1,465	In homozygotes for the -1131 T major allele, fat intake was associated obesity, whereas in those carrying the *APOA5*-1131 C minor allele, higher fat intakes were not associated with higher BMI.	Sanchez-Moreno et al. [40]
*APOE* (4 SNPs)	BMI, WC	Psychological stress	Observational study	Danish men	Obese (*n* = 475), controls (*n* = 709)	The *APOE* rs439401 TT-genotype was associated with an adverse metabolic profile in a population of psychologically stressed Danish men. The TT genotype was associated positively with BMI and WC in stressed men compared with those not similarly stressed	Iqbal Kring et al. [42]
*IRS1* (rs2943641)	Weight loss	Weight loss diets	Clinical trial	POUNDS LOST trial: overweight adults	738	Individuals with the CC genotype might obtain more benefits in weight loss than those without this genotype by choosing a high-carbohydrate and low-fat diet	Qi et al. [62•]
*LCT* (−13910 C > T rs4988235)	BMI, WC	Milk and dairy product intake	Observational study	Spanish individuals at high CVD risk	940	The *LCT* variant was strongly associated with BMI and obesity and its effect was modulated by lactose. CC individuals had lower weight, BMI and WC than T-allele carriers. These associations were found to be significant only among those consuming moderate or high lactose intakes	Corella et al. [45]
*LEP* (5 SNPs)	BMI, weight loss	Low-fat, Mediterranean and low-carbohydrate diets	Intervention study	The 2-y Dietary Intervention Randomized Controlled Trial (DIRECT)	322	Dynamics in leptin concentrations combined with genetic variability in the *LEP* gene may be a predictor of a long-term weight regain following a dietary intervention	Erez et al. [49]
*PLIN1* (11482 G > A, 13041A > G)	BMI, WC, body fat mass, lean mass	Energy-restricted diet	Intervention study	Spanish obese women	78	In response to a 12-wk energy-restricted diet, women carrying the 11482A allele had a lower reduction in WC than non–A allele carriers, suggesting that the *PLIN1* 11482 G > A variant plays a modulating role on diet-induced changes in body fat and energy metabolism in obese women	Ruiz et al. [50]
*PPARγ* (Pro12Ala rs1801282)	BMI, WC, hip, skinfolds	Fat intake, PA	Observational study	Greek children	*n* = 2,102 (1–6 y), *n* = 794 (10–12 y)	The data suggested an age-dependent gene–diet (SFA, TF) interaction: when taking into account the dietary fat intake, the Pro allele homozygotes are at higher risk of increased adiposity	Dedoussis et al. [44]
38 candidate genes (1,444 SNPs)	BMI	Smoking, PA, alcohol consumption, dietary energy intake	Observational study	The Southern Community Cohort Study	1,173 (AA) and 1,165 (Caucasians)	In AAs, significant interactions were observed between smoking and an SNP in *ADIPOR1*, alcohol consumption, and an SNP in *PPARGC1A* and dietary energy intake, and an SNP in *CYP19A1*. In AA, significant interactions were observed between PA and a SNP in *ADIPOR1*	Edwards et al. [46]
15 candidate genes (123 SNPs): the hypothalamic genes	BMI, weight change	Protein intake, dietary GI	Observational case-cohort study	European individuals (Italy, UK, The Netherlands, Germany, Denmark)	5,584	The data suggested that individuals carrying the *NMB* minor allele (G allele) for rs7180849 are more vulnerable to the deleterious effects of a high GI diet in terms of weight gain	Du et al. [48]
21 candidate genes (187 SNPs): cytokines, adipokines, neurotransmitters and transcription factors	BMI, WC, hip	Polyunsaturated fatty acids	Observational study	The second Bavarian Food Consumption Survey	568	SNPs in *IL-2, IL-6, IL-10, IL-18, TNF-α, TNFRSF1A, TNFRSF1B, TNFRSF21, NPY, NPY1R, NPY5R, MC4R, POMC, PPY, PPARγ, PPARγC1A, LEP, LEPR, ADIPOQ* and *RETN* were genotyped. The obesity risk of minor allele carriers significantly decreased with increasing fatty acid content. A reduced obesity risk for minor allele carriers of most variants with high PUFA content in erythrocyte membranes correlated with dietary PUFA intake, except for the SNP of *TNFRSF21, ADIPOQ*, rs2069762 (*IL-2*), rs4833248 (*IL-2* region 5′), and rs10242595 (*IL-6* region 3′). With the latter genes, subjects homozygous for the major allele benefited from an increased PUFA content	Jourdan et al. [47]

*AA* African American; *BF*% body fat percentage; *BMI* body mass index; *CVD* cardiovascular disease; *EA* European American; *GEI* gene–environment interaction; *GI* glycemic index; *PA* physical activity; *PUFA* polyunsaturated fatty acid; *SAT* subcutaneous fat; *SFA* saturated fatty acid; *SNP* single-nucleotide polymorphism; *TF* total fat; *VAT* visceral fat; *WC* waist circumference

In the past year, several intervention studies reported GEIs for variants in the leptin (*LEP*) [49] and the perilipin (*PLIN1)* genes [50] being associated with difference in weight loss in responses to calorie-restricted diets. In contrast, no evidence was found for the effect of variation in the melanocortin-3 receptor *(MC3R)* gene on weight loss after a 10-week dietary intervention with hypo-energetic diets in obese Europeans (*n* = 760) [51].

### GEI Studies for GWAS Genes

The investigation of GEI for obesity-susceptibility loci identified in recent GWAS is thought to be a more useful strategy than the candidate gene approach. The power to detect GEIs for GWAS loci proven to be robustly associated with obesity is very likely to be higher because of the gene’s strengthened causal inference for an interaction [33••]. Among the recent publications examining GEIs of GWAS loci (Table 2), the majority of observational studies evaluated whether dietary components and physical activity interact with variation in the *FTO* gene for their effect on obesity [52–56]. While three studies observed that the effect of the *FTO* risk alleles for obesity was modulated by energy intake or physical activity [52–54], one study with a sample size of more than 6,000 individuals found no evidence for this GEI [55]. The issue was clarified by an impressively large meta-analysis that included data from 45 studies involving 218,166 adults and 9 studies comprising 19,268 children and adolescents [57••]. This meta-analysis confirmed that the minor allele of the *FTO* rs9939609 variant increases the risk of obesity in adults and showed that this risk was reduced among physically active individuals by 27 %. This interaction was more pronounced in North American than in European individuals. The investigators highlighted that their finding is highly relevant for public health implications at the population level (i.e. the individuals with a high genetic susceptibility to obesity can reduce their risk by living a physically healthy lifestyle) [57••]. In addition, a novel finding of the breastfeeding protective effect on the relationship between *FTO* variants and adiposity indices in Greek children from the ages of three upward has been published [58]. A further three papers reported the effect of lifestyle modifications on the relationship between several GWAS genes and obesity-related traits in observational [59] and intervention studies (Table 2) [60, 61].

**Table 2:** Selected gene–environment interaction studies on obesity for GWAS genes

Gene (SNP)	Obesity phenotype	Lifestyle factor	Type of study	Population	Sample size	Major findings	Reference
*FTO* (rs8050136)	BMI	PA, caloric intake	Observational study	Healthy Caucasian women	21,675	The effect of the A-risk allele on BMI was larger among inactive or higher intake women, with additive effects of inactivity and high intake on the associated genetic risk	Achmad et al. [52]
*FTO* (rs9939609, rs1121980)	BMI	PA, fat and carbohydrate intake	Observational study	GOLDN and the BPRHS studies	GOLDIN (*n* = 1,069), BPRHS (*n* = 1,094)	The SFA intake modulated the association between *FTO* and BMI. Homozygotes for the *FTO*-risk allele had higher BMI compared with those with other genotypes only with a high SFA intake. Also, a significant interaction between PA and *FTO* on BMI was found	Corella et al. [53]
*FTO* (rs9939609)	BMI	PA	Meta-analysis	45 studies of adults, 9 studies of children and adolescents	218,166 adults, 19,268 children and adolescents	The association of the *FTO* risk allele with the odds of obesity is attenuated by 27 % in physically active adults, highlighting the importance of PA, in particular in those genetically predisposed to obesity	Kilpelainen et al. [57••]
*FTO* (rs9939609)	Childhood obesity	Dietary fatty acid intake	Observational study	Spanish children and adolescents (6–18 y)	354	Consumption of >12.6 % SFA (of total energy) and an intake ratio <0.43 PUFA:SFA were associated with higher obesity risk in A-risk allele carriers than TT subjects	Moleres et al. [56]
*FTO* (rs9939609, rs17817449)	BMI, WC, skinfolds	Breastfeeding	Observational study	Greek children from the GENDAI and GENESIS studies; British children from the ALSPAC study	1,138 Greek peri-adolescent, 2,374 Greek children 1–6 y, ALSPAC (*n* = 4,325)	A short period of at least 1 month of breastfeeding was associated with reduced obesity indices (WHR, BMI and skinfolds triceps) for the Greek children of different ages homozygous for the rare allele, indicating the breastfeeding protective effect under an obesogenic environment	Dedoussis et al. [58]
16 obesity-susceptibility SNPs	Weight reduction	Weight loss–inducing interventions	Randomized controlled trial	The Diabetes Prevention Program: overweight/obese adults with IGT	3,234	GEI for short-term (6 month) and long-term (2 years) weight loss and weight regain (6 mo to study end). Gene–lifestyle interactions were observed for short-term (*LYPLAL1*; *GNPDA2*; *MTCH2*) and long-term (*NEGR1*; *FTO*) weight loss	Delahanty et al. [63•]
8 obesity-susceptibility SNPs	BMI, SAT	Resistance training program	Intervention study	Young individuals	796	Men carrying the A allele for rs9939609 (*FTO*) lost a significant amount of subcutaneous fat with exercise. Women with a copy of the G allele for rs7498665 (*SH2B1*) showed less change of subcutaneous fat volume after exercise	Orkunoglu-Suer et al. [61]
6 obesity-susceptibility genes (6 SNPs)	Childhood obesity	Sedentary behavior, PA	Observational study	Chinese children (6 − 18 y)	2,848 (1,229 obese cases/1,619 controls)	A higher obesity risk was observed in children who carried the high-risk alleles of the 6 SNPs (in *FAIM2*, *NPC1, FTO*, *MC4R, BDNF*, *GNPDA2*) and engaged in sedentary behavior outside of school or participated in low or moderate PA. The association between 5 genes (*FAIM2, NPC1, FTO, MC4R* and *BDNF*) and obesity risk was only observed in children who had moderate to low PA or engaged in sedentary behavior, regardless of which risk alleles they carried	Xi et al. [59]

*AA*, African American; *BF*% body fat percentage; *BMI* body mass index; *BPRHS* The Boston Puerto Rican Health Study; *GOLDN* The Genetics of Lipid Lowering Drugs and Diet Network study; *CVD* cardiovascular disease; *EA* European American; *GEI* gene–environment interaction; *GWAS* genome-wide association study; *IGT* impaired glucose tolerance; *PA* physical activity; *PUFA* polyunsaturated fatty acid; *SAT* subcutaneous fat; *SFA* saturated fatty acid; *SNP* single nucleotide polymorphism; *TF* total fat; *VAT* visceral fat; *WC* waist circumference

Among the recently published papers, two clinical trials reported GEI in response to weight loss interventions. Qi and colleagues [62•] found a novel association between the variant in the insulin receptor substrate 1 (*IRS1*) gene and response to a weight loss diet: 738 overweight adults (61 % were women) were randomly assigned to 1 of 4 diets varying in macronutrient contents for 2 years. Participants with the *IRS1* rs2943641 CC genotype had greater weight loss and improvement of insulin resistance than those without this genotype in response to a high-carbohydrate/low-fat diet. Interestingly, the variant rs2943650 (*r* ^2^ = 1.00 with rs2943641) near *IRS1* has been reported in a recent GWAS for percentage body fat with the fat percentage–decreasing allele being associated with (counterintuitively) higher levels of insulin resistance [18]. Another study, a randomized controlled trial in overweight and obese adults (*n* = 3,234), investigated the effect of 16 novel GWAS obesity-susceptibility variants on weight loss during a 2-year intervention program. The researchers reported gene–lifestyle interactions for short-term and long-term weight loss [63•]. Altogether, these novel findings provide supportive information for the development of effective dietary intervention strategies based on genetic background.

So far, only one study has examined whether the genetic predisposition to obesity risk assessed by a genetic risk score (GRS) was modified by lifestyle factors. A large-scale population-based study (*n* = 20,430) investigated the effect of a GRS calculated by summing 12 BMI-increasing alleles across the 12 genetic variants and its interaction with physical activity on obesity risk. The researchers found that the genetic risk of obesity was attenuated by 40 % in physically active individuals compared with physically inactive individuals [64••]. These results provide further evidence that particular individuals who are genetically predisposed to obesity would benefit more from elevated physical activity levels than individuals who are genetically protected. Importantly, these findings also indicate that GEIs might contribute to the unexplained variance in obesity traits and suggest that future GWAS of obesity-related traits studying, for example, physically inactive individuals may discover new obesity-susceptibility loci because the effect sizes of genetic variants may be more pronounced and, thus, more easily identified. To our knowledge, numerous consortia-based meta-analyses are ongoing in which this innovative genome–environment-wide association approach is deployed, but so far, no results of these studies have been published.

## Machinery That Underlies GEI

Environment has inarguably a large impact on human physiologic functions and health. Despite the recent successes in identifying genetic determinants accounting for obesity, the definition and quantification of GEIs has proven difficult. Environmental exposure to nutritional and other stimuli can alter the expression of a subset of genes through changes in the epigenome [65]. Although little is known about the exact role of the epigenome in the pathophysiology of obesity, epigenetic regulation of gene expression may be a key factor explaining interindividual differences in adiposity-related phenotypes [66] and the study of the epigenome offers hope in understanding the machinery that underlies complex GEIs.

## Epigenetics

Epigenetics is loosely defined as the study of heritable changes in gene function without modifications in DNA sequences [67]. Epigenetic changes include DNA methylation, chromatin folding and binding, packaging of DNA around nucleosomes and covalent modifications of the histone proteins that make up the nucleosomes around which the DNA double helix is coiled [68]. The epigenome varies across different cell types and undergoes precise, coordinated changes during a lifetime [69••, 70].

DNA methylation is a well-studied epigenetic modification that involves the addition of a methyl (CH3) group to a cytosine located next to a guanine nucleotide (CpG) in CpG dinucleotide–rich regions [70]. Methylation in promoters-associated CpG islands is associated with a transcriptionally repressed state established by two main mechanisms: inability of transcriptional factors to bind to their cognate sequence due to the presence of a methyl group within the binding site or the attraction of methyl-CpG-binding proteins with repressive properties [71, 72].

## Environmental and Genetic Effects on the Epigenome in the Context of Obesity

**Fig. 1:**
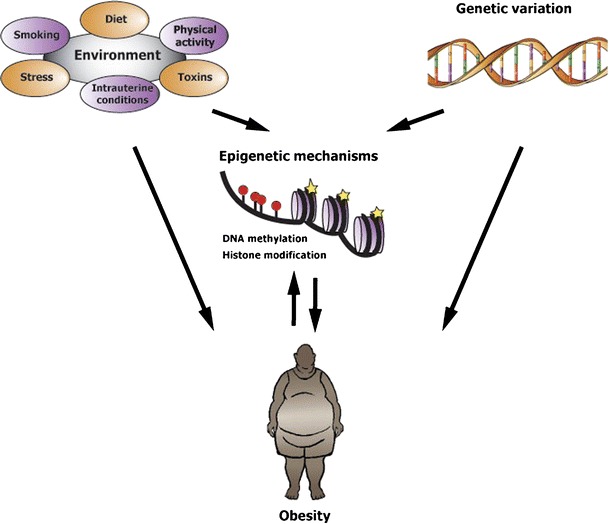
A model of the interplay between environmental/genetic factors and epigenetic changes in the establishment of obesity. Genes, environment, and epigenetic marks can directly lead to increased adiposity. Genes and environment can interact through their influence on the epigenome. Although epigenetic changes may cause obesity, it is often not really clear if they precede obesity, or vice versa

There is increasing evidence of epigenetic regulation of metabolic diseases further supporting a link between genes and environment through their influences on the epigenome [73]. Periconceptional and gestational periods are particularly sensitive to epigenetic perturbation, with the environment exerting different effects on the placenta and embryo [69••]. In particular, nutrition at different developmental stages can influence the epigenome, potentially contributing to an increased susceptibility to chronic diseases, such as obesity [65, 66]. In mammals, early nutrition and in particular dietary components, such as folate, vitamin B6, vitamin B12, betaine, methionine and choline have been associated with changes in DNA methylation patterns by affecting the one-carbon metabolism that ultimately provides the methyl groups for DNA and histone methylation [69••]. Furthermore, maternal food supplementation with bisphenol A—a DNA hypomethylated compound that can leach from polycarbonate plastics into their contents—has been associated with decreased methylation at the A^vy^ allele (the viable yellow agouti allele is a murine metastable epiallele that is variably expressed due to epigenetic marks established during early development) in the offspring and with obesity in early and later life in mammals [68, 74], whereas supplementation of maternal diet with folic acid or genistein negated the hypomethylating effects of BPA [68]. Maternal exposure to several other chemicals (the so-called obesogens) has been associated with increased BMI in offspring, further suggesting that obesity is being programmed prenatally or in early childhood and disruption of normal epigenetic regulation that alters the expression of key genes in adipogenic pathways is likely to be involved [75]. Nevertheless, our understanding of how environmental influence on epigenetic marks can lead to obesity remains rather rudimentary. The potential interaction of environment with the epigenome mediating the expression of genes associated with increased adiposity has also been suggested [76]. For example, the *FTO* gene encodes for an enzyme that is able to remove methyl groups from DNA [77], long-term exposure to high-fat diet can decrease the melanocortin-4 receptor (*MC4R*) gene methylation [78] and high-fat diet–induced obesity can modify leptin methylation patterns [79]. The expression of the *PPARγ* gene, a key regulator of adipocyte differentiation, was found to be reduced due to DNA methylation of its promoter in adipocytes of visceral adipose tissue in mammals [80]. Several other genes involved in adiposity have promoters that seem to be epigenetic targets in relation to obesity (epi-obesogenic genes) [66]. One of the first genome-wide methylation studies revealed increased methylation levels at one CpG site (*UBASH3A* gene) and decreased methylation levels at one CpG site (*TRIM3* gene) in obese subjects compared with lean controls, providing evidence that obesity is associated with epigenetic changes [81•]. Although collectively, these studies could indicate that epigenetic marks lead to obesity, it is not really clear whether they predict or precede obesity [82••]. The causal relationship between epigenetic marks and obesity has yet to be elucidated and other factors, such as nutrition or physical activity, that correlate with both DNA methylation and increased adiposity should be considered to this end [82••]. Genetic differences between individuals can also influence epigenetic regulation [69••] and genetic variants could account for the locus-specific variance in epigenetic states. In humans, it has been shown that 10 % of common SNPs are located in regions with differences in the propensity for local DNA methylation between the two alleles [83]. With this in mind, it is possible that the interplay of genetics and epigenetics could underlie the establishment of diseases, such as obesity. However, the extent to which DNA sequence determines epigenetic changes at specific loci and subsequently leads to obesity is poorly understood. Evaluating the relative contribution of genetic and environmental factors to the establishment of the epigenome and elucidating the causal relationship between epigenetics and obesity constitute major challenges given the complex interrelationship of those determinants (Fig. 1).

## Transgenerational Epigenetic Inheritance

Currently, there is increased evidence that environmentally induced epigenetic changes can also be passed to the next generation via gametes and not only through the placenta in the developing embryo (maternal diet) or through breastfeeding in the infant [84]. Transgenerational epigenetic inheritance is also supported by the fact that some epigenetic marks escape reprogramming—that is, erasure and resetting of the gametic epigenome between generations [73]. This reprogramming escape, in combination with the observation that parental exposure to challenging environments, results in maladaptive responses that can be passed to the next generation renders trasngenerational epigenetic inheritance a mechanism of great interest, especially for obesity. A recent study in mice has examined the effect of a maternal exposure to a high-fat diet on body size, not only in the second generation (F2), but also in the third one (F3) in order to test whether the phenotype is transmitted by a germline-based epigenetic mark. Interestingly, the study has shown that the increased body size and length phenotypes were transmitted to F3 females through the F2 paternal lineage, suggesting that maternal high-fat diet programs a germline-based transgenerational phenotype in male gametes [85]. With this in mind, it is possible that environmentally induced epigenetic changes could in theory explain a significant fraction of the missing heritability for adiposity-related phenotypes by affecting both disease penetrance and heritability [86].

## Challenges of GEI Studies

GEI studies can be very helpful in unraveling the biological pathways important for predicting disease risk and possibly in explaining some of the missing heritability through the identification of obesity-susceptibility genes that exert their effects through interaction with environment. Furthermore, GEIs could potentially be used for the identification of environmental factors that affect individuals with specific genotypes [19••]. However, the investigation of such interactions in complex diseases such as obesity remains a challenging task, with the major limitations including sample size/power, measurement of environmental factors, heterogeneity and lack of replication. Typically, thousands of samples are needed in candidate gene–based studies or even more in GWAS, in which very stringent cutoffs of significance are used. Not all reviewed studies have had a sufficient sample size to detect interactions and the lack of control for type 1 error continues to be a concern. In addition to that, accurate measurement of exposures that vary over time or are modifiable by other factors, such as time of exposure, has proven difficult and can create biases in the analysis. Another important issue is the observed heterogeneity in the study design that arises due to differences in the way that examined environmental exposures are assessed across studies and due to the possible study-specific characteristics of exposure [19••]. Recent efforts in the establishment of prospective cohorts (e.g. the National Children’s Study [http://www.nationalchildrensstudy.gov] and the Avon Longitudinal Study of Parents and Children [http://www.bristol.ac.uk/alspac/]) with robust and repeated measurements over time of environmental exposures can help in assessing the role of critical windows of susceptibility that likely correspond to the expression of specific genes. The challenges related to GEI studies were the topic of discussion in a recent workshop at which more than 150 researchers representing a wide range of scientific areas participated. Interesting questions were raised and useful recommendations were given regarding GEI study design for overcoming the aforementioned limitations. The need for integration of environment, genetics and epigenetics in the same study was also emphasized, as this could provide insight into their complex interactive role in the establishment of disease [87••]. In the post-GWAS era, the careful design of epidemiologic studies, accurate measurement of exposures and use of standardized methods across studies should facilitate collaborations, which will increase statistical power for assessing GEIs. With the completion of the Human Epigenome Project (http://www.epigenome.org/) [88], a more comprehensive picture of the genetic factors and epigenetic marks underlying cellular homeostasis will be achieved. Determination of disease-specific epigenetic changes and integration of this information with genetic and known environmental risks to obesity will provide more insights and will be proven valuable in predicting the onset and progress of obesity.

## Conclusions

Obesity is a complex disease with multiple environmental and genetic causes. Over recent years, the GWAS experimental design has led to the identification of a number of obesity-susceptibility genes that, however, only explain a small portion of the interindividual variation in adiposity. Identifying the genes that predispose to obesity in combination with specific environmental exposures is very important for better understanding of disease etiology and subsequently for disease treatment and prevention. The investigation of gene–environment interplay can also unravel the pathways involved in obesity and be beneficial for drug development and therapy. To date, studies of GEIs have been facing challenges and, thus, are limited compared with those examining only main genetic or environmental effects. Furthermore, the contribution of the epigenome to the establishment of obesity is largely unknown. Further GEI studies that are carefully designed can extend the list of genetic loci that exert effects in the presence of specific environmental exposures. The next generation of studies incorporating genetic–environment–epigenome information and utilizing new analytical approaches and environmental measurement technologies can improve understanding of the complex causes of obesity.
